# Possible Effects of Volcanic Eruptions on the Modern Atmosphere of Venus

**DOI:** 10.1007/s11214-024-01054-5

**Published:** 2024-04-05

**Authors:** Colin F. Wilson, Emmanuel Marcq, Cédric Gillmann, Thomas Widemann, Oleg Korablev, Nils T. Mueller, Maxence Lefèvre, Paul B. Rimmer, Séverine Robert, Mikhail Y. Zolotov

**Affiliations:** 1 European Space Agency, Keplerlaan 1, 2201, AZ Noordwijk, The Netherlands; 2 Physics Dept, Oxford University, Oxford, OX1 3PU UK; 3 LATMOS/IPSL, UVSQ Sorbonne Université Paris-Saclay, Sorbonne Université, CNRS, Guyancourt, France; 4 Institut für Geophysik, Geophysical Fluid Dynamics, ETH Zurich, Sonneggstraße 5, 8092 Zürich, Switzerland; 5 LESIA, Observatoire de Paris, Université PSL, CNRS, Sorbonne Université, Université Paris Cité, 5 place Jules Janssen, 92195 Meudon, France; 6 Université Paris-Saclay, UVSQ, DYPAC, 78000 Versailles, France; 7 Space Research Institute (IKI), Russian Academy of Sciences, Moscow, 117997 Russia; 8 Institute for Planetary Research, DLR, Rutherfordstraße 2, 12489 Berlin, Germany; 9 Institute of Geosciences, Freie Universität Berlin, Malteserstr. 74-100, 12249 Berlin, Germany; 10 Cavendish Laboratory, University of Cambridge, JJ Thomson Avenue, Cambridge, CB3 0HE UK; 11 Royal Belgian Institute for Space Aeronomy, Brussels, Belgium; 12 School of Earth and Space Exploration, Arizona State University, Tempe, AZ 85287-1404 USA

**Keywords:** Venus, Venus evolution, Planetary volcanism, Venus exploration, Volcanic plumes, Detecting volcanism

## Abstract

This work reviews possible signatures and potential detectability of present-day volcanically emitted material in the atmosphere of Venus. We first discuss the expected composition of volcanic gases at present time, addressing how this is related to mantle composition and atmospheric pressure. Sulfur dioxide, often used as a marker of volcanic activity in Earth’s atmosphere, has been observed since late 1970s to exhibit variability at the Venus’ cloud tops at time scales from hours to decades; however, this variability may be associated with solely atmospheric processes. Water vapor is identified as a particularly valuable tracer for volcanic plumes because it can be mapped from orbit at three different tropospheric altitude ranges, and because of its apparent low background variability. We note that volcanic gas plumes could be either enhanced or depleted in water vapor compared to the background atmosphere, depending on magmatic volatile composition. Non-gaseous components of volcanic plumes, such as ash grains and/or cloud aerosol particles, are another investigation target of orbital and *in situ* measurements. We discuss expectations of *in situ* and remote measurements of volcanic plumes in the atmosphere with particular focus on the upcoming DAVINCI, EnVision and VERITAS missions, as well as possible future missions.

## Introduction and Overview

In this paper we review how observations of Venus’ present-day atmosphere place any constraint on the rate or style of volcanic activity in the present era.

One might first ask whether the very nature of the Venus’ atmosphere, in particular its atmospheric pressure (∼47-110 bars depending on elevation) and its thick envelope of sulphuric acid clouds, provides any evidence for volcanism in the present era. In other words, does the current atmosphere represent an equilibrium state which can exist indefinitely without volcanic outgassing? Notably, Bullock and Grinspoon (2001) calculated that the persistence of the sulphuric acid cloud deck could be maintained only through supply of sulphur dioxide gas (SO_2_), presumably through volcanism, within the last 20-50 My. However, such calculations require many assumptions, in particular regarding surface-atmosphere reactions which serve as a sink for atmospheric volatiles (Zolotov 2018). New constraints on surface composition and on volcanic resurfacing styles will provide much needed constraints for assessing the surface-atmosphere exchange, as would the experimental testing of Venus analogue materials, as addressed in companion papers by Gilmore et al. (2023), Ghail et al. (2024), Herrick et al. (2023), and Gillmann et al. (2022). The specific case of using noble gas isotopic ratios such as ^4^He/^3^He to constrain recent volcanism is reviewed by Avice et al. (2022, this collection). There are still too many unknowns today to allow a firm conclusion that the current known atmospheric composition provides evidence of present-day active volcanism, but promising new avenues of investigation are being developed in the coming decade.

Another approach is to search for the transient effects of active volcanism – atmospheric plumes of volcanic gases or particulates – as these may be readily observable. Esposito (1984) suggested that variable SO_2_ abundances observed in the Venus’ mesosphere could be the result of active volcanism. In the past decade, Marcq et al. (2013) showed that these episodic variations of mesospheric SO_2_ continued into the Venus Express years (2006-2014) and proposed that these variations were caused by transient increases of atmospheric mixing between the SO_2_-rich troposphere and SO_2_-poor mesosphere. These fluctuations in vertical atmospheric mixing could be caused by, for example, vertically propagating atmospheric waves (Kouyama et al. 2017), changes in the chemical composition of the cloud particles (Rimmer et al. 2021), variations in static stability profile caused by variations of solar light absorption, or even buoyancy anomalies triggered by volcanic eruptions (Esposito 1984). This, along with interpretations of surface observations in the near infrared spectral range and the Magellan radar images (Smrekar et al. 2010; Shalygin et al. 2015; D’Incecco et al. 2021; Gülcher et al. 2020; Filiberto et al. 2020; Stofan et al. 2016), was interpreted as possibly providing indirect evidence for a recent or ongoing formation of silicate lava flows. However, many key questions about current-day volcanic emissions are still open. What is the redox state and elemental content of volcanic emissions? What is speciation of magmatic volatiles and volcanic gases compared to the atmosphere of Venus? What is the possible range for volcanic gas emissions rates?

Detectability of volcanic products (gases and particulates) in the atmosphere depends on volcanic gas compositions and fluxes, the dimensions, density and dynamics of volcanic plumes, the altitudes and geographical locations of sampling, and the capabilities of analytical instruments on orbiters, descent probes and landers. In addition, detectability will depend on how volcanically emitted species are transported through the atmosphere and how they interact with gases and aerosols, through coupling of chemical and dynamical processes (Bullock and Grinspoon 2001; Wilson et al. 2008; Titov et al. 2018; Lefèvre et al. 2018, 2020).

In this review we will present the different types of atmospheric compositional observations possible from incoming missions to Venus. For each one of these, we will discuss what species can be observed, and what those observations can tell us about the current and/or geologically recent volcanic activity. In Sect. 2, we address the possible composition breakdown and influx rate of magmatic volatiles entering the atmosphere. In Sect. 3, we discuss how volcanic plumes would be detectable with *in situ* instrumental capabilities, such as the DAVINCI mission or future descent probe or aerial platform concepts (Widemann et al. 2023, this collection). In Sect. 4 we discuss remote sensing capabilities, especially how tropospheric gases could be mapped from orbit, as can be carried out by near-infrared spectroscopy (e.g. EnVision/VenSpec-H, EnVision/VenSpec-M and VERITAS/VEM). In Sect. 5 we turn to mapping of gas species in the upper atmosphere, whether through nadir spectroscopy of reflected sunlight, such as EnVision/VenSpec-U investigation. In Sect. 6 we focus on particulate matter: ash particles, sulfuric acid aerosols, or other materials - which might be measured by future *in situ* missions with cloud-level balloons and/or aerial platforms. Finally, in Sect. 7, we suggest directions for further observational constraints and modeling efforts.

## Magmatic Volatiles - Which Gas Species Would Venus’ Volcanoes Emit Today and Which Can We Measure?

### Composition of Outgassed Magmatic Volatiles

**Fig. 1:**
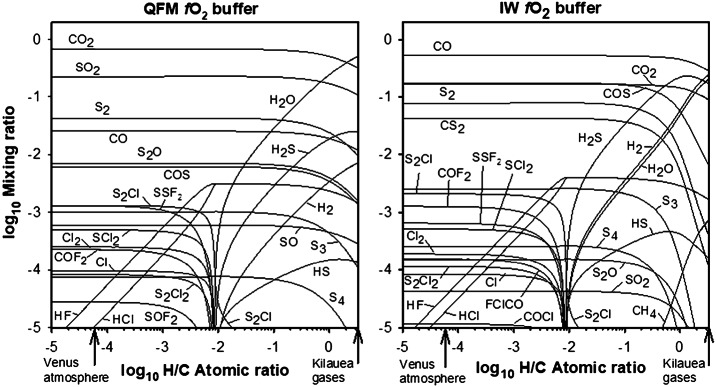
Effects of H content on speciation of volcanic gases at 95.6 bars and 1500 K (modified after Zolotov and Matsui 2002). The elemental composition of gases corresponds to reconstructed 1918 analysis of Kilauea magma lake gases (Gerlach 1980) with variable C/H ratio. Volcanic gases on Earth are enriched in H relative to C (Symonds et al. 1994; Oppenheimer et al. 2014) with the H/C atomic ratio of ∼1 to ∼2000. Kilauea emissions are among the most H-depleted gases. Venus’ counterparts could be more H-depleted than Kilauea gases owing to both H deficiency in the interior and suppressed degassing of high-solubility H_2_O at the ambient pressure of ∼95 bars. The QFM $f$O_2_ buffer corresponds to the quartz-fayalite-magnetite equilibrium and roughly represents the oxidation state of the Earth’s mantle (Frost and McCammon 2008). The IW buffer stands for a more reduced iron-wüstite mineral assemblage. Venus’ basaltic magmas and corresponding magmatic gases could have $f$O_2_ between that of QFM and IW buffers (Schaefer and Fegley 2017). It follows that the figure provides a plausible range of Venus’ volcanic gases.

**Fig. 2:**
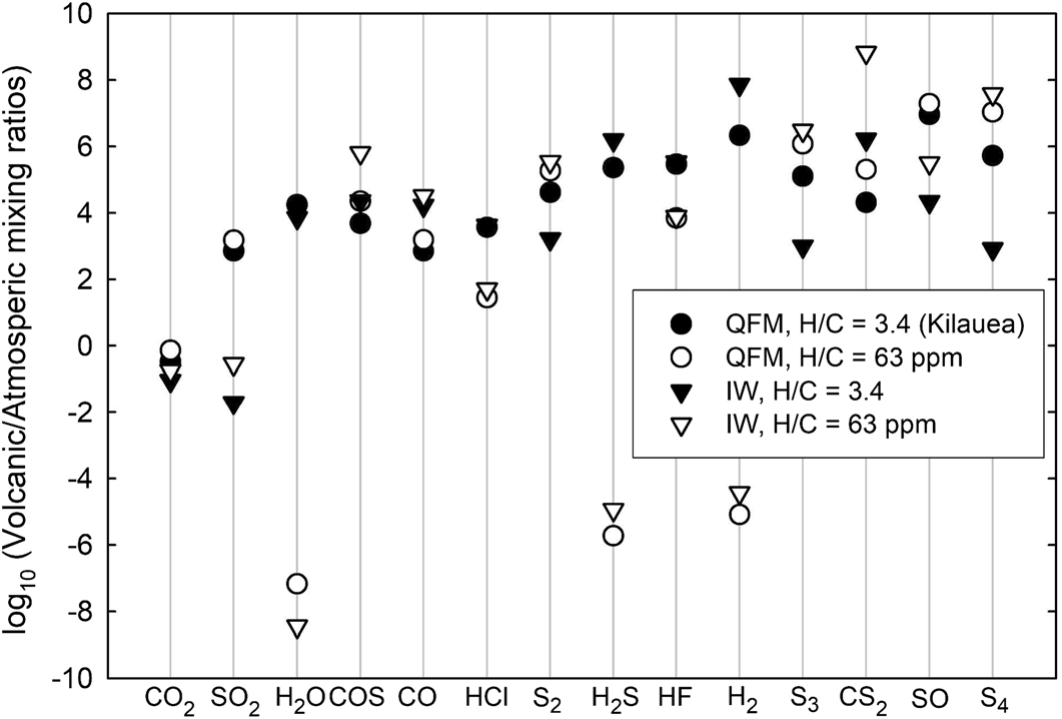
Volcanic/Venus atmospheric gas ratios for Earth-like volcanic gases (filled symbols) and H-depleted gases (empty symbols). Concentrations of volcanic gases correspond to Kilauea-like compositions and H-depleted composition with the atmospheric H/C atomic ratio from Fig. 1. The atmospheric composition corresponds to observations (CO_2_, SO_2_, H_2_O, CO, HCl, HF) and chemical equilibrium models for the near-surface atmosphere (Zolotov 1996). The figure demonstrates that most volcanic species before dilution and chemical interactions could be distinguished from atmospheric gases.

On Earth, chemical footprints of eruptions are often traced by measurements of SO_2_ and other S-bearing gases (e.g., Oppenheimer et al. 2011; Henley and Hughes 2016). The relatively high mixing ratio of SO_2_ in Venus’ lower atmosphere (∼1-2 × 10^−4^) limits the probability of recognizing volcanic sources of this gas, and the same evidently applies to the much more abundant CO_2_. However, our evaluations demonstrate that the compositions of both Earth-like moderately H-rich volcanic gases and putative H-depleted volcanic gases on Venus (Fig. 1) could be distinguishable from the atmosphere (Fig. 2). Locally elevated concentrations of SO_2_, H_2_O, CO, OCS, S_2_, HCl, and HF may indicate volcanic sources. Ratios between these gases will further help distinguish nominal atmospheric features and signatures related to volcanism. Concentrations of these volcanic plume gases would decrease due to dilution through mixing with the air and through chemical interactions. Several orders of magnitude differences between supposed volcanic and atmospheric concentrations of gases (Fig. 2) suggest gas-gas type chemical reactions that consume volcanic COS, CO, S_2_, S_3_, S_4_, CS_2_, SO and exotic S- Cl- F-bearing species that all are out of chemical equilibrium with respect to atmospheric gases. However, reaction kinetics of volcanic and atmospheric gases remains to be assessed and compared with rates of gas mixing and dilution.

**Fig. 3:**
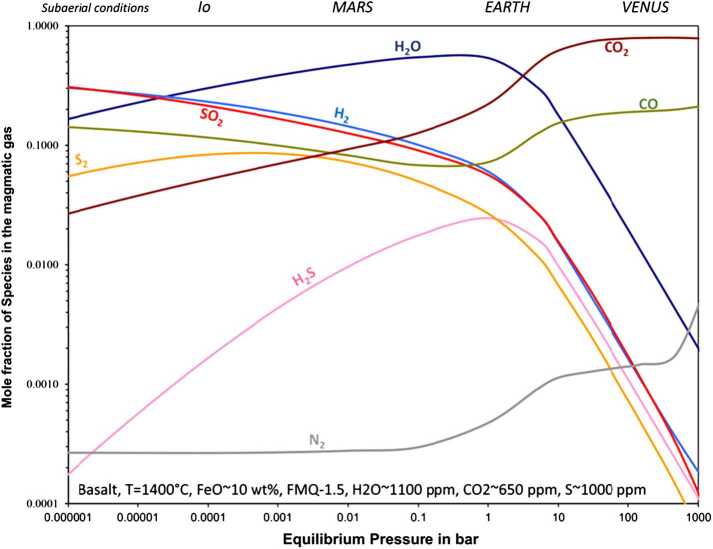
Modeled composition of C-H-O-S bearing volcanic gases with respect to pressure for a given basalt composition. Note that composition of volatiles at the bottom of the figure represents basaltic melt rather than volcanic gases. From Gaillard et al. (2021).

Besides the bulk volatile content (e.g., H/C/S ratios) and oxygen fugacity of parent magmas, the outgassed composition is also expected to depend on the vent pressure that reflects ambient atmospheric pressure (Gaillard and Scaillet 2014). The typical breakdown is shown in Fig. 3. Whereas at low to moderate surface pressures (<1 bar), SO_2_ and H_2_O dominate the mix, it is not the case for high vent and atmospheric pressures. On Venus, low-solubility C-bearing species (CO_2_ and CO) are expected to dominate owing to suppressed degassing of SO_2_ and H_2_O due to their high solubility at elevated pressures. This may make direct detection of volcanic outgassing more challenging, since CO_2_ is the dominant species (∼97vol%) in the atmosphere, and CO a significant species at a background level of a ∼10-20 parts per million by volume (ppmv) in the first scale height. However, direct detection of H_2_O-bearing volcanic plumes on Venus may still be possible despite this pressure suppression, just as detection of SO_2_ plumes is often achieved on Earth despite some suppression of its degassing at 1 bar pressure. Beyond direct detection, though, one also needs to consider post-eruption perturbations to the atmospheric composition resulting in changes in mixing ratio for a vast array of species, as detailed below.

#### CO_2_

As shown in Fig. 3, CO_2_ is likely the most abundant gas in Venus’ volcanic emissions at the Earth-like oxidation state on parent magma (Gaillard et al. 2021; Zolotov and Matsui 2002) – but plumes of volcanic CO_2_ would be indistinguishable from atmospheric CO_2_. The speculations about an endogenic cause of supposedly depleted N_2_/CO_2_ ratio below ∼7 km (Lebonnois and Schubert 2017) would require unrealistically high volcanic CO_2_ flux (Cordier et al. 2019; Lebonnois et al. 2020). Indeed, the apparent age of volcanic plains (0.2-1 Ga, Basilevsky et al. 1997; Korycansky and Zahnle 2005; Bottke et al. 2016) and unconfirmed signs of only very local present volcanism without much pyroclastic activity (Bondarenko et al. 2010; Smrekar et al. 2010; Shalygin et al. 2015; D’Incecco et al. 2021; Gülcher et al. 2020; Filiberto et al. 2020) are inconsistent with a major current CO_2_ flux from the interior. The higher Venus’ CO_2_ atmospheric abundance than Earth’s crust normalized to planetary masses (e.g., Lécuyer et al. 2000) points to degassing of Venus’ interior CO_2_ in previous epochs via volcanism and/or metamorphic decomposition of carbonates formed in putative aqueous environments.

#### CO

A decrease in CO sub-cloud content toward the surface observed by the Pioneer Venus Large Probe and Venera 12 probe (Oyama et al. 1980; Gel’man et al. 1980), telescopic, and orbital (Venus Express) measurements (Esposito et al. 1997; Mills and Allen 2007; Marcq et al. 2018) is explainable by consumption in gas-gas type reactions (e.g., CO → OCS, Krasnopolsky 2007, 2013; Yung et al. 2009). The observed latitudinal differences in tropospheric CO profiles are consistent with the CO to OCS conversion in the deep atmosphere and the Hadley cell type circulation that causes lower CO contents at low latitudes where air is upwelling from the near-surface atmosphere (Tsang et al. 2008; Tsang and McGouldrick 2017). Further understanding of CO and OCS chemical cycles may be revealed by improved mapping of their latitudinal and altitudinal variability through remote sensing, as will be discussed below.

#### H_2_O and HDO

Tropospheric water vapor can be measured remotely in the infrared range at various wavelengths, more than any other trace species, so that its vertical profile is relatively well constrained and consistent with a vertically uniform abundance of ∼30 ppmv at heights of less than 48 km (see Table 1 in Sect. 4.1), pointing to a lack of surface or tropospheric sources or sinks. *In situ* measurements from humidity sensors on board Vega probes (Surkov et al. 1987) exhibited much larger values in the clouds (up to 200 ppmv in the 30-45 km altitude range) - although this measurement may have been contaminated by cloud aerosol particles. In any case, the relatively dry Venusian atmosphere and good remote sensing sensitivity makes tracking of water horizontal, vertical and temporal variations a key observable to constrain any outgassing provided it is water-rich enough. Moreover, gaseous HDO can also be measured, so that D/H ratio is accessible, e.g., through nightside near-IR atmospheric windows between 1 and 2.5 μm, at altitudes 0-45 km (see below, Sect. 4.1) or *in situ* probe measurements (Garvin et al. 2022). Any significantly measured value lower than the background value, (120 ± 40)× Standard Mean Ocean Water (De Bergh et al. 1991; Donahue et al. 1997), would also point to ongoing outgassing from an interior source whose D/H ratio is closer to the solar system average than the value in the bulk Venusian atmosphere, often assumed to have been heavily altered by a strong differential H vs. D escape (Avice et al. 2022, this collection; Gillmann et al. 2022, this collection).

**Table 1:** Summary of post-Venus Express remote measurements of tropospheric water vapor.

Spectral window	Altitude (km)	H_2_O gas abundance (ppmv)	References	Notes
1.10 μm & 1.18 μm	0-15	25-40 25-31 25-30	Chamberlain et al. (2013) Arney et al. (2014) Fedorova et al. (2015)	- retrievals sensitive to assumed surface emissivity - D/H could be measured in principle
1.74 μm	15-30	30-35	Arney et al. (2014)	
2.3 μm	30-45	29-33 30-36	Marcq et al. (2008) Arney et al. (2014)	D/H ≈ 120±40 SMOW (De Bergh et al. 1991)

#### SO_2_

Sulfur dioxide has been measured *in situ* below the clouds from Pioneer Venus and Venera 12 descent probes using mass spectrometry and gas chromatography (Oyama et al. 1980; Gel’man et al. 1980), but the most extensive vertical profile below the clouds was obtained using UV absorption spectroscopy by the ISAV spectrometers on Vega 1 and 2 entry probes (Bertaux et al. 1996). The ISAV data showed a marked decrease in SO_2_ concentration from around 100 ppm at 40 km down to some 25 ppm at 10 km, below which the data become unreliable. Bertaux et al. (1996) suggested that this gradient provided evidence of a strong downward transport of SO_2_, with a SO_2_ sink in the lower atmosphere and/or surface, and SO_2_ source above 40 km, which could be associated with volcanic activity, or recent impacts or cloud processes. However, this gradient and putative sink is difficult to reconcile with known atmospheric processes (Krasnopolsky 2007), and reactions with surface materials are likely too slow to explain such a sink (see the following section). The possible latitudinal variations in SO_2_ content below the clouds (at 30-40 km) (see Fig. 6 in Marcq et al. 2021) and in lower clouds (51-54 km; Oschlisniok et al. 2021) reported from remote sensing looks like an atmospheric phenomenon related to the Hadley cell circulation. Constraining SO_2_ behavior through further remote sensing is discussed below.

#### Hydrogen halides

HCl and HF in the mesosphere have been measured from ground-based and space-based spectroscopy (e.g. Esposito et al. 1997; Mills and Allen 2007). Recently, extensive monitoring by Venus Express using solar occultation appeared to show some variability at altitudes 70-115 km (Mahieux et al. 2015). Below the clouds at altitudes of 30-40 km, though, no spatial variability has yet been found (Iwagami et al. 2008; Arney et al. 2014). It is likely that the HCl and HF are in equilibrium with the surface minerals held at the observed gas-phase concentrations of HCl (0.4 ppm) and HF (2-8 ppb) (Fegley et al. 1992). The low Cl/S and F/S atmospheric ratios compared to possible volcanic gases could be explained by a more efficient consumption of volcanic halogen-bearing gases to secondary surface minerals, and the low concentrations of HCl and HF indicate past gas-solid reactions that led to gas-solid equilibration (Zolotov 2018). Aside from putative fluctuations in HCl and HF contents just below the main cloud level, any positive compositional anomalies in the sub-cloud atmosphere could be indicative of current volcanism. Detection of such anomalies could be achieved by remote observation in the 1.74 μm spectral window or by multiple atmospheric profiles from entry probes.

#### PH_3_

The claimed detection of phosphine (Greaves et al. 2021), has been debated in recent years and has been proposed as a possible tracer of volcanism (Truong and Lunine 2021; Omran et al. 2021); but this PH_3_ purported signature is difficult to reconcile with other non-detections (Encrenaz et al. 2020b; Villanueva et al. 2021) as well as current oxidized (Bains et al. 2021) and dry (Bains et al. 2022) atmospheric composition. Other phosphorus species could constrain mantle composition and volcanic rates, and a combination of these species can provide a loose constraint on mantle redox state, under the assumption that these species are volcanically derived.

### Surface and Atmospheric Chemistry Involving Trace Volcanic Gases

In contrast to atmospheric processes, slow gas-solid type reactions in a permeable upper layer (e.g., Fegley et al. 1992; Fegley et al. 1997a,b; Wood 1997; Zolotov 2018) cannot cause gradients of CO, SO_2_, or other atmospheric gases. Atmospheric mixing through eddy diffusion and turbulence (e.g., Krasnopolsky 2007; Morellina and Bellan 2022) in the near surface atmosphere and the Hadley cell circulation are much faster than gas-solid reactions. The thermodynamically favorable oxidation of ferrous minerals (silicates, FeS, Fe_1-x_S) and glasses in basalts produce CO at the expense of CO_2_ (Zolotov 2018) as illustrated by simplified reactions that are irrelevant to the CO atmospheric gradient:

$$\begin{aligned} &3 \text{FeSiO}_{3} \text{ (ferrous pyroxene)} + \text{CO}_{2} \rightarrow \text{Fe}_{3}\text{O}_{4} \text{ (magnetite)} + 3 \text{SiO}_{2} \text{ (silica)} + \text{CO}\\ &3 \text{FeS (troilite)} + 4 \text{CO}_{2} \rightarrow \text{Fe}_{3}\text{O}_{4}\text{ (magnetite)} + \text{CO} + 3 \text{OCS} \end{aligned}$$

These and other oxidation reactions, especially further oxidation of magnetite to ferric oxide hematite (Fe_2_O_3_), could be limited because the atmospheric CO/CO_2_ ratio is indistinguishable from that controlled by the magnetite-hematite equilibrium (Fegley et al. 1997b; Zolotov 2018). In contrast to CO, atmospheric SO_2_ is consumed through reactions with Ca- and Na-bearing phases in basalt leading to formation of sulfates and S_2_ gas (Zolotov 2018):

$$\begin{aligned} \text{CaSiO}_{3} \text{(Ca silicate)} + 1.5\text{SO}_{2} \rightarrow \text{CaSO}_{4} \text{(anhydrite)} + \text{SiO}_{2} \text{(silica)} + 0.25 \text{S}_{2} \end{aligned}$$

Feasibility of the latter interactions on Venus has been illustrated by modeling experiments (e.g., Fegley and Prinn 1989; Berger et al. 2019; Radoman-Shaw et al. 2022; Santos et al. 2023). However, trapping of SO_2_ would likely be limited by equilibration with sulfate- and/or pyrite-bearing mineral assemblages and because of inefficient or inhibited alteration of plagioclase (Zolotov 2018).

It is possible that volcanic gases would be depleted in hydrogen, for Venus (Zolotov and Matsui 2002), Io (Fegley and Zolotov 2000), and Mercury (Zolotov 2011; Evans et al. 2015). The H-depleted volcanic gases could not have abundant H_2_O, H_2_S, HCl, and HF (Fig. 1). Chlorine, fluorine and partially sulfur and carbon are presented by OCS, CS_2_, S_2_, S_3_, S_4_, S_2_O, S_2_Cl, S_2_F_2_, SO, SCl_2_, Cl_2_, Cl, COF_2_, SOF_2_, and S_2_Cl_2_. With the exception of S_3_ and S_4_ (Maiorov et al. 2005; Krasnopolsky 2013), and OCS (see Mills and Allen 2007; Marcq et al. 2018 for reviews), none of these gases have been detected in the sub-cloud atmosphere. Any detection of these gases below clouds could therefore be indicative of current volcanism (Fig. 2). However, these gases are thermodynamically unstable in contact with lower atmospheric gases and could be converted into HCl, HF, Cl- and S-bearing gases. Likely consumption of Cl- and F-bearing gases through net hydrolysis reactions

$$\begin{aligned} &\text{SCl}_{2} + 2 \text{H}_{2}\text{O} \rightarrow 2 \text{HCl} + \text{SO}_{2} + \text{H}_{2} \\ &\text{Cl}_{2} + \text{H}_{2}\text{O} + \text{CO} \rightarrow 2 \text{HCl} + \text{CO}_{2} \end{aligned}$$

will deplete atmospheric water vapor and increase concentration of the reaction products (e.g. HCl) that could be observed in a partially diluted volcanic plume. Reaction kinetics of H-free volcanic gases remain to be evaluated for Venus conditions. Although the consumption lowers chances of detection in diluted plumes away from a vent, near-vent sampling of H-depleted gases from a descent probe or a lander would be more informative than for H-rich gases (Fig. 2). Such a detection will strongly constrain water history and water content in the interior of the planet.

### Possible Volcanic Processes and Outgassing Rates

The volcanic production rate on Venus, and the associated outgassing rate are still poorly constrained. Using an equilibrium resurfacing model based on the age of the surface, Phillips et al. (1992) obtained a rough estimate of ≃ 1 km^3^/yr, consistent with Earth-like hotspot production rates. Namiki and Solomon (1998) find that ^40^Ar and ^4^He abundances in the atmosphere of Venus are consistent with magmatism rates in the order of 1-4 km^3^/y, but highlight high uncertainties. Stofan et al. (2005), based their estimate on the volume of lava required to bury impact craters since the emplacement of the present-day surface and found a range of 0.6-46 km^3^/yr (we note though that the high end of this range is for an improbably surface age of only 10 Myr). Romeo and Turcotte (2010) propose a post-catastrophe resurfacing rate of ∼ 2 km^3^/yr and note that equilibrium resurfacing models they tested resulted, by contrast, in global present-day resurfacing rates of > 40 km^3^/yr to explain the statistics of the observed crater population. Ivanov and Head (2013) estimated the volume of volcanic units on Venus and derive resurfacing production rates between 0.03 and 0.1 km^3^/yr depending on the age of the surface (300 Myr to 750 Myr).

Byrne and Krishnamoorthy (2022) scaled the rates of volcanism from Earth to Venus using the planets’ masses and volumes and estimated 120 eruptions per Earth year on Venus. However, van Zelst (2022) took into account tectonic difference associated with volcanism and estimated only 12 per year. Other estimates are based on the outgassing of SO_2_ necessary to sustain the current SO_2_ abundance in the clouds, and yield values of 0.3-11 km^3^/yr (Fegley and Prinn 1989) and 4.6-9.2 km^3^/yr (Bullock and Grinspoon 2001). However, two latter evaluations are reliant on poorly known composition of the near-surface atmosphere and may benefit from being revisited based on future data. Both evaluations assume an existence of Ca carbonates in surface materials that is not possible based on SO_2_-calcite interaction experiments (e.g., Fegley and Prinn 1989; Santos et al. 2023), evaluations of stability of Ca carbonates with respect to S-bearing atmospheric gasses, and the lack of mechanism for carbonate formation at the current surface conditions (e.g., Zolotov 2018).

The degassing rates will depend on the dynamics of the mantle; and the connection between these two has been explored by e.g., Noack et al. (2012), Gillmann and Tackley (2014). One should note that a stagnant lid regime limits the efficiency of heat loss from the mantle. It has been calculated that under such conditions, the interior of Venus may heat up and cause widespread melting, where volcanism is used to extract heat to the surface in a heat-pipe regime (e.g. O’Reilly and Davies 1981; Moore et al. 2017). Volcanic production rates consistent with this scenario are in the order of 170-280 km^3^/yr, an order of magnitude above Earth’s total production (Solomon and Head 1982; Armann and Tackley 2012). Short episodes of intense resurfacing separated by long periods of relative quiescence or a predominantly intrusive emplacement of melt appear to be more consistent with suggested Venus’ present-day heat flux and estimations of crustal thickness. The complex links between melt production and outgassing have been briefly mentioned above in Sect. 2.1, and are discussed in greater detail by Gillmann et al. (2022), for example.

Other papers in this collection address the expected styles of volcanism as evidenced by their morphology and occurrence. Ghail et al. (2024, this collection) describe the large diversity of volcanic features with generally no direct analogs in Earth’s volcanism. Among volcanic features, large volcanic rises or hotspots may be directly related to mantle plumes, whereas features such as coronae, or extremely long lava channels require more detailed observations to be understood. Coronae have been used to suggest (Ghail et al. 2024, this collection) that most of the magma delivery on Venus would be intrusive (Lourenço et al. 2020), because heat pipe models, in which all mantle heat is extracted through extrusive volcanism, predict crustal production rates that would produce a much younger surface than observed (Moore et al. 2017; Rolf et al. 2022). Geophysical considerations of (Head and Wilson 1986, 1992) suggested abundant magma chambers in a zone of neutral buoyancy in the Venus’ crust.

The style of volcanic eruption will affect the detectability of volcanic gas plumes, in that it will affect both how episodic an emission of gases is, and to what altitude the plume of gases will rise. Of particular importance is the volatile content of the erupting magma: not only does this determine whether an eruption will be explosive, injecting gases high into the atmosphere, but would also be an important indicator of the long-term evolution of the interior (Rolf et al. 2022, this collection; Gillmann et al. 2022, this collection). Explosive volcanism would throw not only gas but also ash into the atmosphere, likely expressed on the surface in the form of pyroclastic deposits (Head and Wilson 1986; Airey et al. 2015; Ganesh et al. 2021); a few suspected instances of pyroclastic deposits have been found on Venus (Campbell et al. 2017; Ganesh et al. 2022; Ghail and Wilson 2015; Keddie and Head 1995). The volatile content needed to produce an explosive eruption varies with the barometric pressure and therefore with vent altitude. For a suspected pyroclastic flow feature identified at Scathach Fluctus, it has been calculated that the volatile content needed to produce an explosive eruption here must be relatively high: 4.5 weight percent (wt%) if only H_2_O, or 3% H_2_O plus 3% CO_2_. On the other hand, explosive volcanism at the summit of Ma’at Mons would require only 2% H_2_O content (Airey et al. 2015). A thin layering of apparently porous rocks seen as low flat outcrops with darker soil in between them at Venera 13 and 14 landing sites could have formed through airborne deposition of impact-generated particles (Basilevsky et al. 2004, 2019), though a low viscosity lava crystallization phenomenon (Garvin et al. 1984) and volcanic pyroclastic origins (Florensky et al. 1983) are possible. Such deposits could bring evidence regarding the volatile content of lava flows, possibly reflecting an early stage of renewed magmatic activity with volatile-rich, disrupted magma escaping through vents in fractured regions of the upper crust (Campbell et al. 2017). Herrick et al. (2023, this collection) notes that future InSAR missions (see the review in Widemann et al. 2023, this collection) will significantly improve our understanding of the sequence of events on the surface and evaluate whether or not fundamental changes in the nature of geologic activity have occurred over the past several hundred million years.

The above discussion has focused on explosive volcanism largely because it would likely be the easiest to detect, due to the large gas release in a short period of time and possible higher altitude reached by the ensuing volcanic gas plume. Much of the discussion of volcanic gas detection is equally applicable to effusive (non-explosive) eruptions or even from passive outgassing (i.e. involving no coincident eruption of magma). Determining whether the style of eruption is effusive or explosive would help to constrain magmatic volatile content, with implications for the evolution of Venus. This could be achieved either by looking for surface deposits like pyroclastic flows, or by detection of ash plumes in the atmosphere, as discussed below.

### Vertical Transport of Volcanic Gases

In Sect. 2.3 we have presented a discussion of volcanism styles and outgassing rates, which will determine the source characteristics of a volcanic plume. We now discuss the subsequent transport of volcanic gases in the atmosphere. We will first discuss the vertical transport of plumes because this is essential for determination the vertical profiles of volcanic gases in the atmosphere.

The elevation that a volcanic plume can eventually reach is determined by the temperature of the volcanic gases and by the atmospheric temperature-pressure profile: as long as the rising gas plume remains of lower density than the surrounding atmosphere, despite its own adiabatic cooling as its barometric pressure decreases, then it will continue to rise. The canonical view is that volcanic gas plumes typically cannot overcome the convective stability of the current atmosphere of Venus, with the exception of particularly large eruptions (Glaze 1999; Glaze et al. 2011; Airey et al. 2015). However, the above works (in particular the first two) were motivated largely by the question of whether volcanic plumes would rise to the cloud-tops at 70 km or above, to explain mesospheric SO_2_ variations from space such as those from Pioneer Venus Orbiter UV measurements (Esposito 1984). A rising plume would require a lot of buoyancy to rise through the convectively stable layers found at altitude ranges of 35-50 km and 60-70 km. However, reaching 30 or 35 km altitude is comparatively much easier: the atmospheric temperature profile appears to be near zero stability at 0-10 km and 20-35 km altitude, indicating that plume rise in these altitude bands could be readily achieved even with a small excess temperature (on the order of only 10 K).

The above discussion is based on the mean atmospheric temperature profile of the Venus International Reference Atmosphere (VIRA) – (Seiff et al. 1985), and therefore comes with two important caveats. The first is that this temperature profile is subject to significant uncertainties, particularly in the deepest 12 km. None of the Pioneer Venus probes returned usable temperature data below 12 km, so the VIRA profiles were based largely on the Venera 10 probe profile, which itself had significant uncertainties. The most reliable temperature profile is from the Vega 2 probe, but this appears to show a superadiabatic (convectively unstable) layer at 0-7 km altitude which is yet unexplained (Lebonnois and Schubert 2017; Lorenz et al. 2018; Morellina et al. 2020; Morellina and Bellan 2022). New measurements of temperature and composition near the surface from DAVINCI descent probe (Garvin et al. 2022) will reduce these uncertainties.

The second caveat about using the VIRA mean profile is that it will vary spatially and diurnally; using a mean profile ignores these variations and also neglects the effects of atmospheric dynamics. Lebonnois et al. (2018) found that the diurnal cycle of the planetary boundary layer convection is expected to be influenced by topography. Above large topographic features, the diurnal cycle of the convective layer is expected to be quite strong, extending up to 7 km above the local surface at noon and dropping below 1 km at night. Such strong vertical mixing would enhance the vertical propagation of the plume material. The low convective stability of the boundary layer in the late afternoon conditions would also enable the propagation of topographically-driven waves from the surface up to the cloud tops, as discussed by Lefèvre et al. 2020. Follow-up observations by Akatsuki LIR camera and modeling lend further support to these findings (Lefèvre 2022; Suzuki et al. 2023).

## In Situ Measurements of Volcanic Gases in the Troposphere

Having presented an overview of possible volcanic gases of interest, of styles of volcanism, and of vertical transport of volcanic gases in the atmosphere, we now turn to focus on measurements from future missions, starting with *in situ* descent probe measurements in Sect. 3 before turning to orbital remote sensing measurement in Sect. 4. The discussion is based on the DAVINCI probe, as it is, at the time of writing, the only confirmed future mission which will measure gas composition *in situ* in the atmosphere. Other future candidate missions will be discussed in Sect. 5 below.

### Expected Constraints on Volcanism from the DAVINCI Mission

NASA’s DAVINCI mission (Garvin et al. 2022; Widemann et al. 2023, this collection) is scheduled to sample chemical and isotopic compositions in the lower atmosphere in early 2030s with the Venus Mass Spectrometer (VMS) and the Venus Tunable Laser Spectrometer (VTLS). Although these instruments have different performance capabilities, they will provide data on mixing ratios of major reactive gases (CO_2_, SO_2_, H_2_O, CO, OCS, H_2_S, S_n_, H_2_SO_4_, and HCl) and stable isotope composition of H, O, C, S, He, Ne, Ar, Kr, and Xe. The VMS is capable of measuring gases in the range of 2-535 Da. Measurements by VMS and VTLS will constrain abundances of P-bearing gases that may be present in the atmosphere but may not be volcanic (Krasnopolsky 1989; Baines et al. 2021). Sampling of reactive gases will occur every 0.15-1 km at altitudes ranging from ∼11 to 50 km, and more often (50-100 m) below ∼11 km. Stable isotope data for H, O, C and S will be obtained through at least 5 VTLS measurements below 40 km. The D/H ratio will be measured with 2% precision up to ten times from 67 km to ∼2 km. Abundances of noble gases (He, Ne, Ar, Kr, Xe) and N_2_ and their major isotopes will be measured by the VMS at least once below 70 km. The precisions are as follows, ^4^He (5%), ^40^Ar (15%), ^136^Xe (20%), ^129^Xe (20%). Major atmospheric and purported volcanic gases are certainly detectable with these instruments. Accuracies of measurements (SO_2_, 5%; H_2_O, 20%, CO, 5%; OCS, 3%), will allow identification of moderately diluted and/or chemically altered plumes. Gases emitted by putative H-depleted melts (CS_2_, SCl_2_, Cl_2_, etc., Fig. 1) are within the mass range of the mass spectrometer, though evaluations and tests are needed to assess their detectability and detection limits.

On Earth, volcanic gases (Symonds et al. 1994; Oppenheimer et al. 2014) are isotopically distinct from the air, and expected measurements from the DAVINCI mission of vertical profiles of ^16^O/^18^O, ^12^C/^13^C, and ^32^S/^33^S/^34^S would be informative to distinguish a plume. Locally low D/H ratios will indicate current degassing because the high atmospheric D/H ratio is likely resulted from a preferential H escape (e.g., Donahue et al. 1997). Locally elevated ^40^Ar/^36^Ar ratio will indicate current degassing because ^40^Ar accumulates in the interior through decay of ^40^K. On Earth, the ^3^He/^4^He ratio in volcanic gases is used to distinguish interior source regions (e.g. Graham et al. 2002; Hilton et al. 2002), and a local ^3^He/^4^He anomaly in the lower atmosphere could place constraints on magmatic sources of volatiles. Note that these constraints could be obtained only with multiple sampling of Ar and He at different altitudes that is not currently planned on DAVINCI for the sub-cloud atmosphere. Overall, several correlated compositional (SO_2_, CO, OCS, H_2_O, etc.) and isotopic anomalies of H, O, C and S might be sufficient to catch a volcanic plume in the sub-cloud atmosphere.

In addition to putative current volcanism, DAVINCI expected data on bulk atmospheric ^40^Ar content will constrain both the scale and history of Venus degassing (Kaula 1999; Namiki and Solomon 1998; O’Rourke and Korenaga 2015; Gillmann et al. 2022, this collection; Avice et al. 2022, this collection). Likewise, both early and overall degassing rate could be evaluated from abundances of ^129^Xe and ^136^Xe that are products of radioactive decay in the interior (Coltice et al. 2009). The atmospheric escape rate of helium is on the order of hundreds of millions of years (Fedorov et al. 2011; Nordström et al. 2013), so the bulk atmospheric helium isotope ratios measured by DAVINCI will help to constrain volcanic fluxes on these timescales (Namiki and Solomon 1998). Taken together, these constraints on degassing throughout history from isotopic abundances and ratios will place new bounds on the probability of current and future volcanism on various timescales. In particular, these data will help to distinguish between the alternative catastrophic (Schaber et al. 1992; Strom et al. 1994) and equilibrium resurfacing models that imply approximately continuous and current volcanism (Phillips et al. 1992; Herrick and Rumpf 2011; Bjonnes et al. 2012). Further discussion of noble gas isotope geochemistry and its constraints on recent volcanic outgassing is presented by Avice et al. (2022) in this collection.

The planned DAVINCI deep atmosphere probe descent over Alpha Regio, which represents a strongly tectonically-deformed region (tessera) without clear signs of volcanism (e.g., Bindschadler et al. 1992), decreases the probability of direct (localized) sampling of a volcanic plume. Descent imaging of the Alpha surface from the probe in the near-infrared range and at 0.74 to 1.03 μm will provide new geomorphological information about the prospects of volcanic activity at the touchdown region at spatial scales as fine as meters. With the single atmospheric profile, it will be difficult to unequivocally distinguish atmospheric and volcanic causes of any measured compositional phenomenon, if any are detected. Geographical locations of purported current or recent volcanism (Bondarenko et al. 2010; Smrekar et al. 2010; Shalygin et al. 2015; D’Incecco et al. 2017; Stofan et al. 2016; Gülcher et al. 2020; Filiberto et al. 2020) may need to be selected for future sampling of volcanic gases and particulates by descent probes and landers.

## Orbital Detection of Tropospheric Gas Plumes

In this section we turn to the detection of volcanic gas plumes from orbital remote sounding. We focus at first only on detection of H_2_O-rich plumes; this is because water vapor is detectable from both orbit and ground-based telescopes using nightside near-IR spectroscopy and can be mapped at several altitudes from the surface up to 30-40 km altitude, as will be detailed further below. The discussion in Sect. 2.1 showed that the H_2_O content of volcanic gases is not known on Venus; volcanic gas from an H-depleted magma might well be depleted in H_2_O compared to the atmosphere. Nevertheless, the discussion of plume transport in Sect. 4.1 is applicable to any passive tracer, and can be scaled to any positive or negative water excess, so could even be applied to the transport of H_2_O-poor volcanic plumes. In the second half of this section, we will then extend the discussion to consider other volcanic gas species whose tropospheric abundances could be mapped from orbit.

### H_2_O Tropospheric Plumes

The background abundance of water vapor in Venus’ atmosphere is close to 30 ppmv below the clouds (e.g., Bézard and de Bergh 2007; Marcq et al. 2018; Ignatiev et al. 1997; Pollack et al. 1993). Remote sensing of tropospheric water is possible on the night side using the thermal emission in a few near infrared windows of relative CO_2_ transparency (Taylor et al. 1997), as summarized in Table 1.

Water vapor abundances are expressed in volume mixing ratio (VMR), in units of parts per million by volume (ppmv).

As can be seen in Table 1, the vertical profile of water vapor is uniform in the probed range within the sensitivity of existing measurements. Some hints of horizontal variation of H_2_O have been reported in the 2.3 μm band (sensitive to 30-45 km altitude) by Arney et al. 2014, and from Venus Express/VIRTIS observations (Haus et al. 2015), typically at the 10%-20% level; however, it is not clear whether these are associated with cloud ‘ghosting’ effects in the retrieval as discussed in Arney et al. (2014) and Barstow et al. (2012). No spatial variations in water vapour retrieved in the 0-15 km and 15-30 km altitude windows have yet been found (see e.g., Bézard et al. 2011; Haus et al. 2015). In order to assess the detectability of a plume of volcanic gases, one needs to consider whether it could alter gas abundances by an amount greater than the measurement sensitivity.

**Fig. 4:**
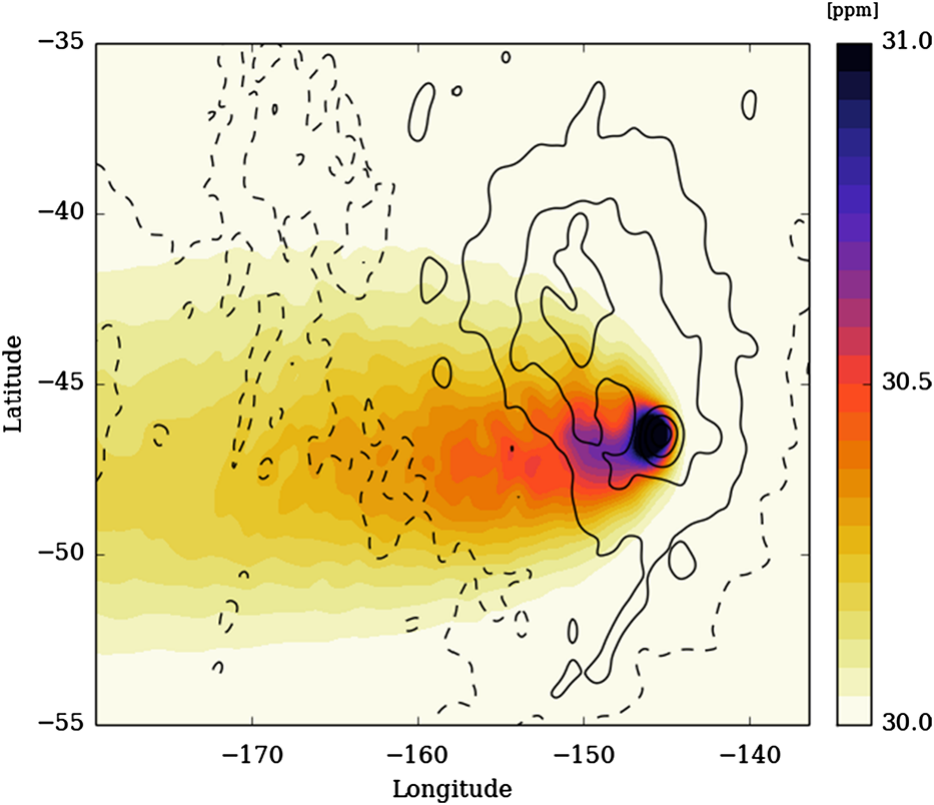
Simulated dispersion of a volcanic plume released from Idunn Mons in Imdr Regio. Shading indicates water vapor abundance at 13 km altitude, contours indicate surface topography. See text for further details of the simulation.

Preliminary mesoscale simulations of atmospheric plume advection were performed by Lefèvre et al. (2020). Outgassing was simulated by setting a constant 1.1 multiplicative factor for H_2_O (33 ppmv instead of 30 ppmv) in a single 10 km × 10 km (surface) × 10 km (height from surface) atmospheric cell over Idunn Mons in Imdr Regio (this is one of the sites of suspected recent hotspot volcanism identified by Smrekar et al. (2010). The thermochemical lifetime of H_2_O below the clouds far exceeds the simulation duration. Results after 10 Earth days are shown in Fig. 4. Thermal buoyancy above the first 10 km is not considered yet, only advection by the atmospheric circulation is. The altitude reached by the plume depends sensitively on the thermal structure of the planetary boundary layer; Lebonnois et al. (2018) showed that this would be highly variable, with high-altitude slopes giving rise to particularly deep convection potentially allowing volcanic gas plumes to reach greater height. Similar modeling is necessary at the equator, for example at the potentially active region found in *Atla Regio* (Shalygin et al. 2015), to study the variability of the deep atmosphere stability and the possible transfer of volatiles between northern and southern hemispheres.

The effective H_2_O emission rate in this simulation is about 30 t/s (2.6 Mt/Earth day) during the simulated 10 Earth days. Such an amount is typical of ‘mid-sized’ eruptions on Earth such as the Bárðarbunga-Holuhraun (Iceland) eruption in 2014 (Schmidt et al. 2015), assuming a H_2_O:SO_2_ mass ratio of 20. The maximal observable enhancement near the source is about 0.8 ppmv, which is marginally below the ppm-level accuracy of water vapor measurements as of 2021. It should however be detectable from future dedicated instruments such as VenSpec-M and -H channels (Helbert et al. 2019) on board EnVision as well as VEM on board VERITAS (Helbert et al. 2018, 2020).

The modelling presented here is preliminary; further investigations of the effects of buoyancy and atmospheric thermal structure, eruptive style and location are needed. However, this approach shows how the detectability of atmospheric plumes from volcanic eruptions of different sizes and styles can be modelled and evaluated. We note that there may also be non-volcanic sources of H_2_O variability near the Venus cloud deck, such as strong convective downdrafts as modelled by Baker et al. (1998), or evaporating H_2_SO_4_ rain (virga) as discussed by Arney et al. (2014) and modelled by Gao et al. (2014); these must be modelled in order to understand potential ‘false positives’ in the hunt for volcanic plumes.

Radiative transfer numerical simulations show that with a high spectral resolution (R∼8000) and a signal-to-noise ratio of 100, VenSpec-H will determine the abundances of H_2_O with an accuracy of 3% in each of the spectral windows centered at 1.18, 1.74 and 2.3 μm (Robert et al. 2021). As shown in Table 1, these three windows allow mapping of water vapor in three different altitude domains, comprising 0-15 km, 15-30 km and 30-45 km respectively.

As well as seeking evidence for plumes (and providing new information about tropospheric dynamics) by mapping the spatial variations of water vapor, VenSpec-H will also search for variations in the HDO/H_2_O ratio. For HDO, preliminary spectral modeling shows that it should be possible to reach an accuracy of 5% in HDO retrievals in the 1.18 μm and 2.3 μm windows, assuming only the random error, i.e. the retrieval error directly from the fit (Robert et al. 2021; ESA 2024). Taking into account also the H_2_O retrieval accuracy described above, this would result in an accuracy of 8% in mapping spatial variability of the D/H ratio between 0 and 15 km and between 30 and 45 km.

### Other Tropospheric Gas Plumes: SO_2_, CO, HCl and OCS

Besides H_2_O, several other gases may be related to present volcanic activity as described in above Sect. 2.1. In particular, tropospheric SO_2_, CO, HCl and OCS could be detected from the orbit, also using nightside IR-spectroscopy. The 2.3-2.5 μm region is therefore targeted as each of these species are known to absorb in this spectral range. Their detectability is not an easy task as the spectral lines are weak, moreover confirming current volcanic activity requires assessing their background (non-volcanic) variability. In addition, the vertical profiles of OCS and CO are known to have anti-correlated behavior at ∼35 km, which could be explained by reactions (Yung et al. 2009; Pollack et al. 1993; Krasnopolsky 2007), that do not require volcanic gases.

**Fig. 5:**
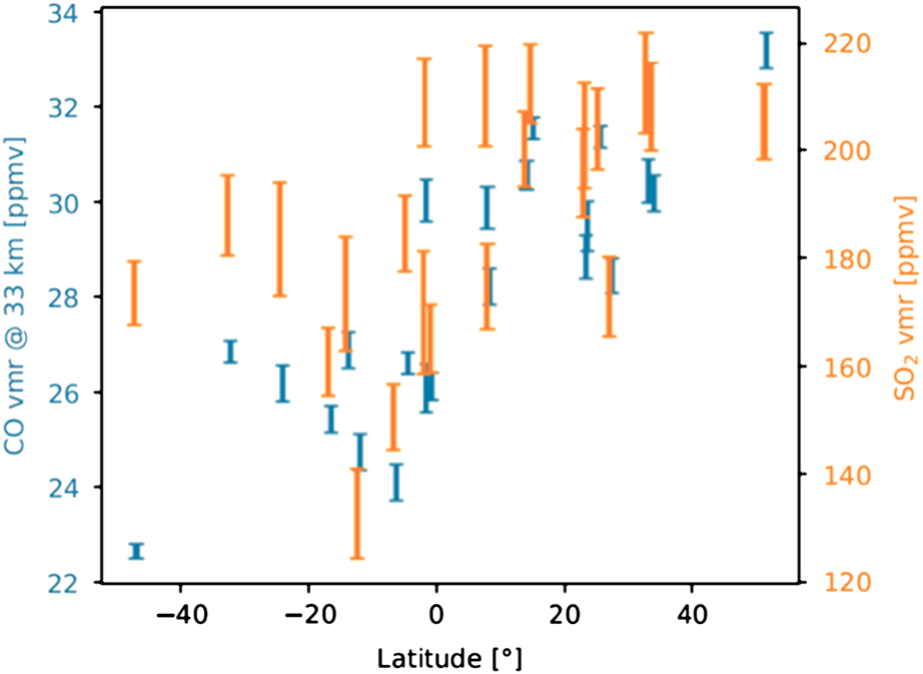
Latitudinal variations of CO and SO_2_ volume mixing ratios at altitude of 33 km are both measurable using remote sounding, as was demonstrated by Marcq et al. (2021) using IRTF/iSHELL. Error bars stand for ±3$\sigma $ standard deviations. Systematic uncertainties of the absolute SO_2_ mixing ratio are not displayed.

Measurements of tropospheric SO_2_ have been performed from Venus Express orbit (Marcq et al. 2008, 2023; Oschlisniok et al. 2021) and from ground-based facilities (Pollack et al. 1993; Arney et al. 2014), leading to an accepted value of 130±50 ppmv in the 30-40 km altitude range (or, in the latest analysis of VIRTIS-H data by Marcq et al. 2023, 190±50 ppmv). The relatively low spectral resolution of the VIRTIS-H spectrometer aboard Venus Express (R∼1800, or more accurately 1500-2000, see Marcq et al. 2008, their Fig. 1) means that SO_2_ retrievals from that instrument reach a precision of only ± 20% or so; at this sensitivity, no evidence of spatial variability was found. More recent observations were performed by Marcq et al. (2021) using the high-resolution iSHELL spectrometer at the NASA IRTF facility (R∼20,000); thanks to this higher resolution, the sensitivity to tropospheric SO_2_ achieved in the retrieval was typically < 5%. With this higher sensitivity, Marcq et al. (2021) were able to show evidence of latitudinal variations of SO_2_ abundances (see Fig. 5). This demonstrates the importance of high spectral resolution for enabling measurement of tropospheric SO_2_ variability, as will be performed by VenSpec-H on EnVision.

Carbon monoxide is also absorbing in the 2.3 μm range between 30-40 km in altitude. CO is already known to increase with increasing latitude (Marcq et al. 2008, 2021, 2023; Tsang and McGouldrick 2017), as shown in Fig. 5, resulting from the large-scale meridional circulation and positive vertical gradient due to its photochemical source above the clouds (Yung and Demore 1982). CO is indeed considered as a passive tracer in Venus’ atmosphere. The results of the numerical radiative transfer simulations are encouraging as an accuracy of 1.5% will be achieved with VenSpec-H (Robert et al. 2021; ESA 2024).

**Fig. 6:**
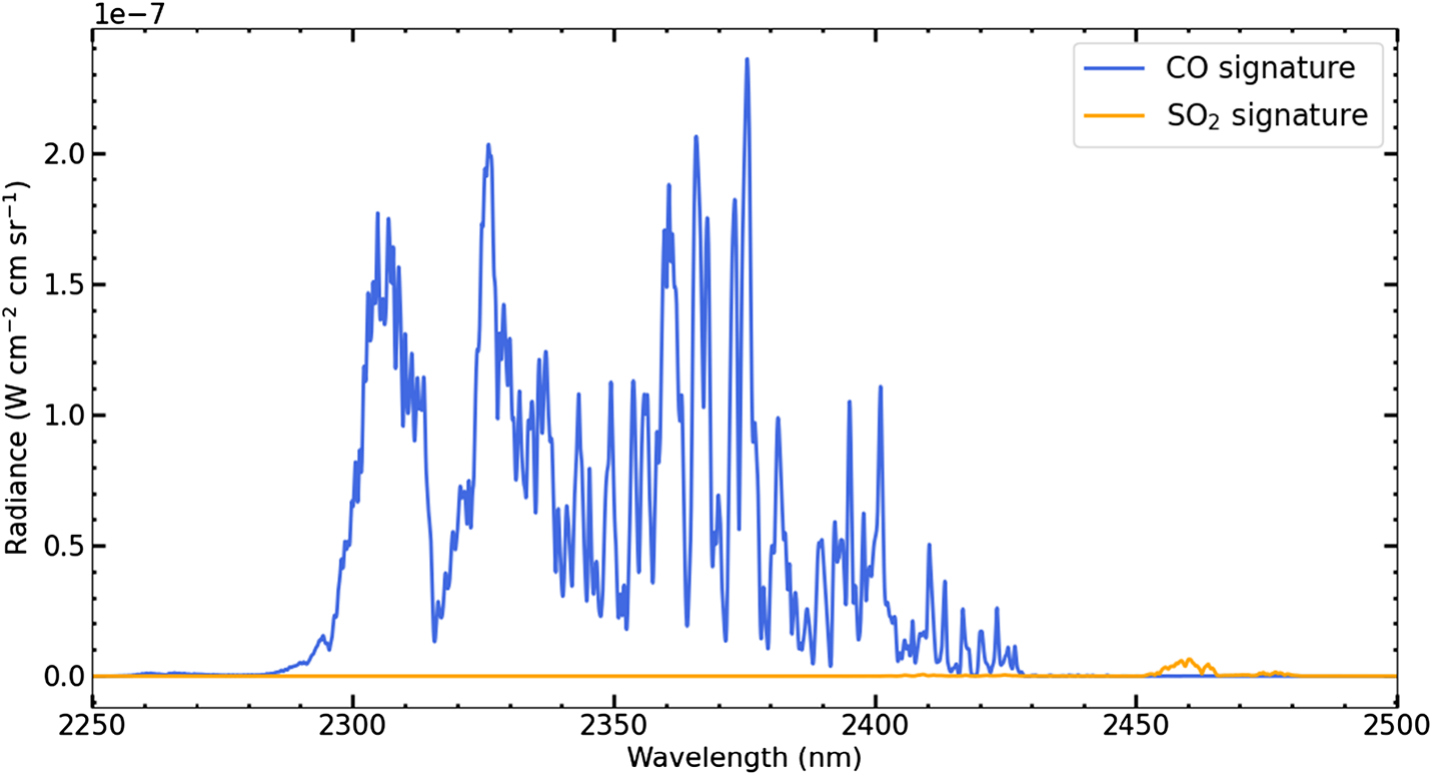
Spectral signatures of CO and SO_2_ in emission from the nightside of Venus in the 2.3–2.4 μm spectral band. The figure shows the difference in spectrum if CO and SO_2_, respectively, are removed from the model atmosphere. Simulations are obtained using the ASIMUT-ALVL radiative transfer tool (Vandaele et al. 2006).

Robustly assessing the presence of a volcanic gas plume necessitates measuring multiple tracer species simultaneously. Therefore, the VenSpec-H instrument has been designed to allow simultaneous measurement in both of the CO and SO_2_ bands as shown in Fig. 6 (as well as H_2_O, HDO, HF, CO, COS, which are not shown; Robert et al. 2021).

We have focused here (in Sect. 4.2) on the 2.3 μm spectral window. In addition, the other nightside transparency windows may bring insights on the current volcanic activity of Venus. HCl, for instance, may be measured in the 1.74 μm window together with H_2_O, probing the 15-30 km altitude range. Investigating further the information contained in the 2.3 μm window necessitates new mesoscale simulations considering the thermal buoyancy to properly address how these trace species (SO_2_, SO, HF) could be used as tracers of present-day outgassing.

## Detecting Volcanic Gases in the Mesosphere

SO_2_ exhibits the most dramatic variations at Venus’ cloud top, both spatially and temporally (Esposito 1984; Esposito et al. 1988; Marcq et al. 2013; Vandaele et al. 2017a,b), spanning more than two orders of magnitude on timescales ranging from a few days up to several decades. Esposito (1984) suggested that the observed episodic variations in mesospheric SO_2_ might be caused by episodic volcanic activity. The SO_2_ injections into the upper atmosphere need not be volcanic SO_2_: instead, the thermal energy and dynamical effects of a volcanic eruption could lead to tropospheric (SO_2_-rich) air being transported into the mesosphere. However, episodic injections of SO_2_-rich tropospheric air into the mesosphere might also be associated with dynamical cycles of the atmosphere, rather than due to volcanism (as argued for example by Marcq et al. 2013). To address this question, EnVision will measure supposed volcanic species both below the clouds (using near-IR nightside spectroscopy as discussed above), and above the clouds (using near-IR and UV spectroscopy on the dayside). If correlations are found between mesospheric and tropospheric gas abundances (or indeed with surface indicators of volcanic activity), that would provide valuable information on how volcanic volatiles are transported through the atmosphere.

**Fig. 7:**
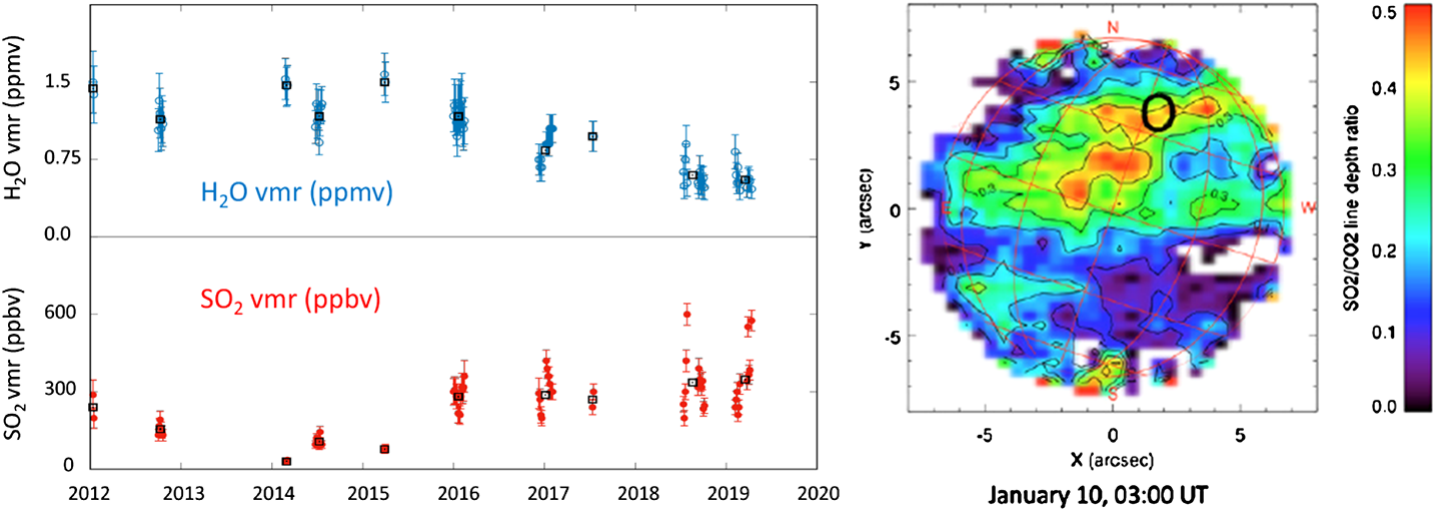
Left: Long-term variations of the H_2_O volume mixing ratio (top, blue points) and the SO_2_ volume mixing ratio (bottom, red points), measured at the cloud top from the TEXES data at 7.4 μm. From Encrenaz et al. (2020a, their Fig. 11); Right hand side panel: SO_2_:CO_2_ line ratio map obtained by TEXES at 7.4 μm by Encrenaz et al. (2012), showing the large instantaneous spatial heterogeneity of SO_2_ near cloud top.

Anti-correlated variations of H_2_O (or its proxy HDO) in the same altitude range also exist, albeit less spectacular and restricted within one order of magnitude (Encrenaz et al. 2020a) as can be seen in Fig. 7. The greater range of SO_2_ variations compared to H_2_O is currently explained by the fast photochemical destruction of SO_2_ by UV sunlight at cloud tops, making this species a much more sensitive tracer of the atmospheric circulation and vertical mixing between its lower atmospheric source (below the clouds, SO_2_ is more abundant by 3-4 orders of magnitude, e.g., Oyama et al. 1980; Gel’man et al. 1980; Bertaux et al. 1996; Pollack et al. 1993; Arney et al. 2014) and its cloud top photochemical sink.

The variations of the vertical mixing are themselves poorly understood and most likely depend on the interplay between atmospheric waves breaking, solar radiative forcing, thus varying with latitude, local solar time and underlying topography. For example, the thickness of the convective layer in the lower cloud layer varies with latitude and local solar time (Tellmann et al. 2009; Imamura et al. 2017), and convective activity near the subsolar point at cloud top level is suggested by observations (Titov et al. 2012) and modeling (Lefèvre et al. 2018). However, the statistical distribution of observed SO_2_ “plumes” by TEXES exhibits a minimum near the subsolar point between 10:00 and 14:00 LST (Encrenaz et al. 2019), as does SPICAV-UV (Marcq et al. 2020). The other main sulfur oxide species, namely SO, could be measured using high resolution UV spectroscopy from HST/STIS (Jessup et al. 2015) and appears to be correlated to SO_2_ with an average ratio SO:SO_2_ of about 10%.

Interestingly, the observed SO_2_/H_2_O anticorrelation at cloud tops (Fig. 7) was predicted by chemical modelers (Parkinson et al. 2015; Shao et al. 2020), on the grounds that both species can be the limiting progenitor species of the cloud droplets made of mostly sulfuric acid. Unfortunately, no chemical model is currently able to quantitatively reproduce both H_2_O and SO_2_ profiles below and above the clouds simultaneously (Bierson and Zhang 2020) – most models focus on either below or above the clouds and tune their lower/upper boundary conditions accordingly. This points to unknown processes operating in the cloud region, involving an extra sulfur reservoir beyond SO_2_ and the sulfuric acid in the droplets. This unknown reservoir may be linked to the unknown UV absorber (which would be a sulfur-based compound in this hypothesis). The most recent hypothesis (Rimmer et al. 2021) postulates a sulfate salt reservoir in order to solve this long standing issue, and will be further discussed in Sect. 6.

**Fig. 8:**
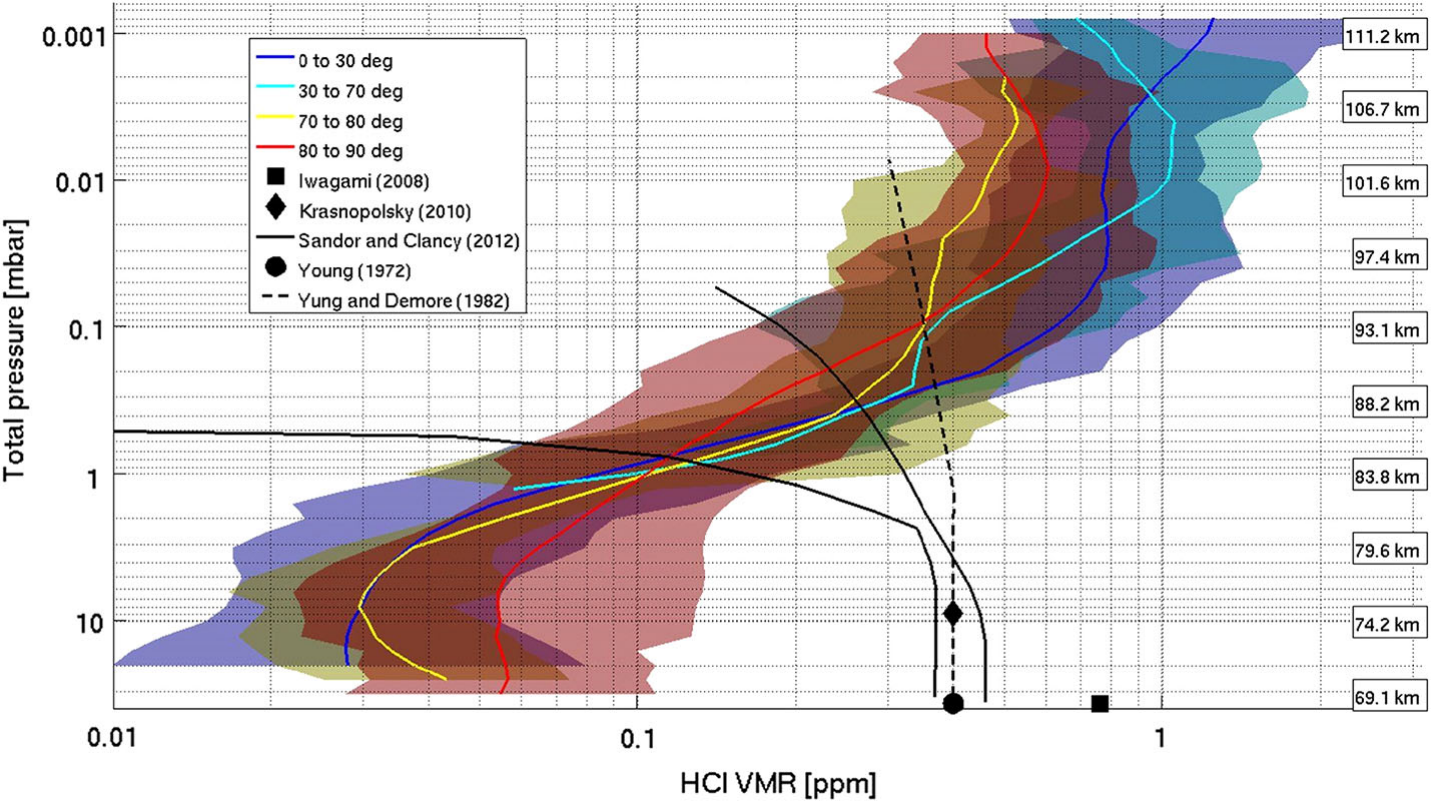
Mean value of the HCl profile obtained by SOIR for different latitude bins as a function of total pressure. The altitude is also shown in correlation with the pressure scale. The weighted standard deviations are the colored shaded envelopes. The profiles are compared to literature data (models + other observations), hinting at a discrepancy in the vertical gradient. From Mahieux et al. (2015, their Fig. 5).

Other possible gaseous species potentially linked with volcanism are harder to monitor above the clouds, since their relatively lower abundance makes them only detectable using the very sensitive solar/stellar occultation technique. This technique strongly constrains the vertical profiles of such species, but the drawback is a poor horizontal and temporal coverage compared to nadir measurements (and, in the case of solar occultations, restricted to 6AM/6PM local solar time except at the very highest latitudes). Nevertheless, the SPICAV/SOIR instrument onboard Venus Express was able to record the first vertical profiles of HCl and HF gases above the clouds in the infrared domain (Mahieux et al. 2015), which showed a great spatial and temporal variability as well as yet poorly understood positive vertical gradients — not seen by other observers, e.g. Sato and Sagawa (2023) — hinting at yet unknown sources/sinks (Fig. 8). In the near future as of 2023, using the same observation technique, the VIRAL instrument onboard ISRO’s planned Venus Orbiter Mission Shukrayaan-1 will also be able to monitor the vertical profiles of the above mentioned species (hydrogen halides, hydrogen isotopes, sulfur oxides) as well as yet unmeasured other species involved in the sulfur chemistry, including H_2_S and OCS at a ppb accuracy level (Patrakeev et al. 2022; Widemann et al. 2023). The expected improvement in measurement sensitivity for mesospheric species from upcoming missions is listed in Table 2.

**Table 2:** Expected measurement accuracy for the volume mixing ratios (VMR) of mesospheric trace species with the instrument VIRAL onboard ISRO’s Venus Orbiter Mission Shukrayaan-1 (Patrakeev et al. 2022; Widemann et al. 2023) and the instruments VenSpec-U and -H onboard ESA’s EnVision spacecraft.

Species	Altitude range (km)	Current VMR measurement accuracy (or upper limit)	Expected VMR measurement accuracy (or upper limit)
H_2_O	70-110	∼1 ppm (Fedorova et al. 2008)	≤0.3 ppm (VIRAL)
	70	∼2 ppm (Cottini et al. 2012)	≤0.2 ppm (VenSpec-H)
HDO	70-95	∼0.1 ppm (Fedorova et al. 2008)	≤30 ppb (VIRAL)
	70		≤20 ppb (VenSpec-H)
HCl	70-110	0.1-1 ppm (Mahieux et al. 2015)	≤30 ppb (VIRAL)
		30 ppb (Krasnopolsky 2010)	
	70	0.379 ± 0.013 ppm (Sato and Sagawa 2023)	
HF	70-100	5-50 ppb (Mahieux et al. 2015)	≤1 ppb (VIRAL)
	70	0.3 ppb (Krasnopolsky 2010)	≤1 ppb (VenSpec-H)
SO_2_	70-80	50-500 ppb (Belyaev et al. 2008; Mahieux et al. 2015)	≤30 ppb (VIRAL)
		10-1000 ppb (Marcq et al. 2013, 2021)	≤30 ppb (VenSpec-H)
	70		≤5 ppb (VenSpec-U)
SO	70	∼1 ppb (Jessup et al. 2015)	≤0.5 ppb (VenSpec-U)
H_2_S	∼70	<23 ppb (Krasnopolsky 2008)	≤3 ppb (VIRAL)
OCS	∼70	∼few ppb (Mahieux et al. 2023)	≤0.3 ppb (VIRAL)
			≤1 ppb (VenSpec-H)

Carbon monoxide may be a major constituent gas in volcanic gas emission, due to the high Venus surface pressures (Fig. 3). It is readily observable in the mesosphere, both in nadir observations (e.g. Irwin et al. 2008) and in solar occultation (Vandaele et al. 2016). However, CO is not usually considered as related to volcanism above the clouds, since its main source in the upper atmosphere comes from the photolysis of CO_2_ (Marcq et al. 2018; Yung and Demore 1982).

In conclusion, variability in many mesospheric trace gas species has already been observed, but it has not yet been possible to attribute any of this variability to volcanism. The EnVision mission will seek to address this by continuing mesospheric and tropospheric composition measurements while simultaneously searching for ongoing volcanic activity on the surface.

## Clouds and Particulates

We previously have considered gaseous species whose detection, whether by *in situ* or remote sensing, could provide evidence for volcanism. Now we turn instead to non-gaseous species: first we consider whether mapping from orbit could reveal plumes of sulphuric acid cloud droplets formed due to volcanically emitted gases and/or plumes of volcanic ash particles; then we consider whether measurements of cloud droplet/particulate composition from an *in situ* cloud exploration platform could reveal about volcanism.

A large volcanic emission of H_2_O or SO_2_ could lead to increased formation cloud droplets, and therefore increased optical thickness and lower radiances in nightside observations of near-IR thermal window regions. To a first approximation, an upper bound on this effect can be reached by assuming that all the volcanic volatile species emitted at the volcano are transported to the clouds and are all turned into cloud particulate matter. For example, if we assume the same Bárðarbunga-Holuhraun 2014 eruption as discussed above, in which about 28,000 kg/s of water are released (Schmidt et al. 2015), this could lead to up to 28,000 kg/s of water droplets being produced (or up to 152,000 kg/s of sulfuric acid cloud droplets being produced if there is sufficiently abundant SO_3_ to combine with. If this were deposited in a layer at 50 km altitude at the base of the clouds in a plume 100 km wide, and taking into account the mean wind velocity of 60 m/s at this altitude (from VIRA), we can calculate that the additional cloud mass in a column would be in the range 5-30 g/m^2^. This is an order of magnitude less than the vertically integrated columnar mass of cloud droplets, of 150 g/m^2^ (as reported in Ragent et al. 1985, Table 3-4), and significantly less than the factor of 2× over which the lower cloud optical depth is observed to vary (Titov et al. 2018). This suggests that the direct formation of aerosols from condensing volcanic volatiles could only be detectable for larger (>100,000 km^2^) eruptions. We emphasize that this is a very simplistic order-of-magnitude calculation, neglecting dynamical and chemical interactions.

Explosive eruptions may inject not only gases into the atmosphere, but often also ash particulates; for example, the 2010 Eyjafjallajökull eruption is estimated to have ejected at least $7\times 10^{10}\text{ kg}$ of fine ash aerosol (characteristic radius < 28 μm) into the atmosphere (Gudmundsson et al. 2012). Peak emission rates of fine ash in that eruption exceeded 40,000 kg/s (Flanner et al. 2014), so of a similar order of magnitude to the volatile release scenario considered above. Volcanic ash aerosols on Earth may have a single scattering albedo at 2 μm of around 0.9 (Mortier et al. 2013), which is much less than the > 0.99 single scattering albedo typical of the Venus cloud particles, so volcanic ash would be expected to have a much greater extinction effect per columnar unit of mass than sulfuric acid aerosol. On the other hand, the residence time of ash particles in the clouds may be much shorter, depending on their interaction with cloud microphysical cycles. We note that remote sounding in nightside IR channels would not be able to unambiguously distinguish between the spectral signatures of ash or cloud droplets in lower cloud, because of the limited number of spectral windows in which observations can be made and the degeneracy between effects of particle size and composition (Wilson et al. 2008). If volcanically lofted particulates reach the clouds, they can provide nucleation sites for condensation of sulfuric acid, which in turn may dissolve silicate material of volcanic ashes, and therefore could change the gas-phase (Rimmer et al. 2021) and solid/liquid phase (Zolotov et al. 2023) composition in the cloud layer. However, mechanisms of delivery and fluxes of volcanic particulates to the clouds remain unknown.

*In situ* measurements of particulate composition at cloud level offer a much more capable method of searching for evidence of volcanism. The most sophisticated *in situ* measurement of cloud composition was made by Venera 12 (Petryanov et al. 1981) and then by the Vega 1 and 2 descent probes: they analyzed the composition of collected cloud & aerosol matter using mass spectrometry, gas chromatography and X-ray fluorescence, complementing measurements of particle size and refractive index from nephelometry. The original data from this impressive suite of instrumentation were not archived. The original analyses, published in Russian-language journals, are summarized by Krasnopolsky (1989) and reviewed by Titov et al. (2018). Of particular note, is a tentative measurement by the X-ray fluorescence spectrometers of significant abundances of phosphorus in the lower cloud particles. The molecular form of this phosphorus is not determined by the instrument; Krasnopolsky (1989) proposed it to be in the form of phosphoric acid. Results of X-ray fluorescence and mass spectrometers (Venera 12, Vega 1 and 2) also suggested chlorine in the cloud particles. Iron in cloud aerosols reported based on Venera 12 X-ray fluorescence spectrometry could be in Fe(III)-bearing salts (Krasnopolsky 1985, 1989, 2017; Zolotov et al. 2023). No evidence of any volcanic ash or other surface materials was reported from entry probe mass spectrometers, but this is not conclusive because such materials would dissolve in concentrated sulfuric acid. Minerals that survived the dissolution likely not have been volatilized when the captured aerosol samples were heated to 400 °C for mass spectrometric analysis. A recent reanalysis of Pioneer Venus Large Probe mass spectrometer data suggests thermal decomposition of trapped cloud particles below cloud deck to the surface, and some captured particles could have formed through chemical alteration of surface-delivered grains in the clouds (Zolotov et al. 2023).

For future cloud-level platforms, an aerosol mass spectrometer (AMS) has been proposed which could analyze the composition of both atmospheric gases and cloud/aerosol particulates (Baines et al. 2021). This would be deployed on a balloon-borne gondola which, during a mission duration of two months, be carried by Venus’ super-rotating winds to complete at least ten complete circumnavigations of the planet, mapping cloud composition as a function of local time, latitude. Baines et al. (2021) propose to deploy such an instrument on an altitude-controlled balloon which would explore altitudes ranging from 52 km (in the lower convective cloud) up to 62 km (in the convectively stable upper cloud). Just as was described in the proposed DAVINCI investigation (see Sect. 3.1), a Venus aerobot investigation of cloud-level particulate and gas composition would search for species which are out of chemical equilibrium with the environment; it would search for “volcanosignature” gases and also seek to characterize the liquid/solid phases with which they are reacting (Cutts et al. 2021; Byrne et al. 2022). In particular, a cloud-level balloon platform with a lifetime of many weeks offers the opportunity to characterize the background environmental and chemical conditions; marked changes to this baseline composition (such as increases in sulfate aerosols) could be indicative of volcanic activity (see also Widemann et al. 2023, this collection).

Such an instrument could also directly search for volcanic ash aerosol, whether this is directly lofted to altitude by a volcanic explosion or remobilized from the surface by wind. Distinguishing current ‘fresh’ volcanic and wind-delivered silicates dust should be possible because ancient volcanic materials could be chemically altered through gas-solid type reactions at the surface (Zolotov 2018). Chemical analysis of dust particles could be performed through the X-ray fluorescence method and/or mass spectrometry with laser ablation, for example. Phase composition could be performed via X-ray diffraction method. Even if this were not possible, detection of volcanic ash particles through nephelometry is another viable possibility; volcanic ash particles are typically highly fractal and therefore exhibit very different phase functions compared to liquid (and thus spherical) cloud droplets. Note that glassy silicate ash particles could dissolve in ∼75% sulfuric acid in hours to days; in that case, any detection of such particles would imply currently ongoing volcanic ash production. Chemical analysis of liquid aerosols could inform initial composition of dissolved particulates. In particular, relative contributions of dissolved cosmic dust (Zolotov et al. 2023) and surface-sourced volcanic materials in liquid cloud aerosols could be assessed.

The discussion here about measurements of particulate composition to be made from a balloon platform would also apply to a descent probe, although the target species would be somewhat different. Descent probes have reported the existence of hazes below the cloud base (Titov et al. 2018), where temperatures are well above the boiling point of sulfuric acid cloud droplets. These hazes, therefore, cannot be composed of sulfuric acid. Similarly, Grieger et al. (2004) reported that Venera descent probe photometry suggested a discrete layer of particulates at 2 km above the mean planetary radius, whose nature is not known and might also be related to volcanic pyroclastic processes (see Carter et al. 2023, this collection).

## Summary and Conclusions

### Further Needed Observational Constraints

Since the exact composition of the possible outgassing depends on the poorly constrained physical and chemical conditions of the magmatic phase, we should refrain from any premature assumptions and look for any possible sign of assessed deviation relative to the baseline abundances of minor species. Time-variability of some out-of- background gas abundances could provide evidence of volcanic degassing, even if it is not definitive.

Remote detection of present-day volcanic outgassing should therefore rely on assessed imbalance over as many simultaneous measurements of detectable trace gases species as possible. In the troposphere, high spectral resolution spectroscopy of the night side thermal emission windows in the CO_2_ spectrum would provide accurate measurements averaged over a horizontal extent of ∼30 km due to multiple scattering in the overlying clouds. HCl, HF, CO, OCS, SO_2_, H_2_O and HDO can be constrained this way below the clouds despite the very high pressure (and even at multiple altitude levels for H_2_O and HDO, with the additional insight provided by the D/H isotopic ratio variations). Incidentally, this same night side thermal infrared spectroscopy is also sensitive to surface mineralogy (through emissivity measurements), and can constrain chemical weathering of surface materials via gas-solid type reactions. Above the clouds, in the mesosphere, evidence for volcanic outgassing would become indirect, and rely on the enhanced sensitivity of some minor species caused by the competition between photochemistry and convective variability (whose origin may be related to ongoing volcanic activity if any). Diagnostic species in this altitude range are therefore photochemically active short-lived species, first and foremost SO_2_ and SO, then H_2_O and HDO, as well as CO or hydrogen halides (and their deuterated isotopes) to a lesser extent. These species have already been detected or even monitored, either through UV spectroscopy or near infrared spectroscopy over Venus’ dayside. These measurements would also benefit from simultaneous observations in the sub-millimeter range of their vertical profiles in the upper mesosphere, providing upper boundary conditions.

Compared to remote sensing observations, *in situ* measurements will be of paramount interest to constrain atmospheric layers that cannot be observed otherwise. Gas chromatographs, mass spectrometers and tunable laser spectrometers on descent probes or more long-lived aerial platforms within the clouds would probe, among other species, volcanogenic volatiles such as sulfur-bearing and halogen-bearing species. Descent probes reaching the surface would be able to measure vertical gradients in the first atmospheric scale height above the ground and could indicate active sources or sinks. On the other hand, dedicated payload on board aerial platforms such as nephelometers will better characterize aerosols and constrain their compositions, including possibly volcanogenic particulate matter (e.g. ashes). Although it is beyond the scope of this paper to discuss it in more detail, we note that infrasound sensors mounted on cloud-level balloons could allow detection of pressure waves from explosive volcanic eruptions, providing a direct measurement on the rate and style of volcanic activity (Rossi et al. 2023). Finally, one of the main objectives of surface landers will be characterization of the surface composition and mineralogy through various means (X-ray fluorescence, X-ray diffraction, Mossbauer spectroscopy, infrared spectroscopy, etc.), which would in return bring constraints on magma composition and/or surface-atmosphere reactions discussed in Sect. 2.

### Further Needed Modeling and Experimental Efforts

Improvements in existing numerical models are already being considered to support future observations described above. Chemical models, including heterogeneous chemistry between the cloud droplets and gases (e.g. Rimmer et al. 2021), are necessary to elucidate the unknown sulfur reservoir issue (Marcq et al. 2018). Coupling these chemical models with dynamical models is also an undergoing effort as of 2023, with projects ranging from general circulation models coupling to mesoscale models (Lefèvre 2022), particularly suited to investigate buoyancy- and dynamically-driven plume dispersal reviewed in this paper’s Sect. 4, along with disequilibrium chemical reactions between the background atmosphere and the volcanogenic volatiles brought by the plume.

Modeling efforts also include laboratory simulations. Some experimental setups are already operational to study alteration of surface minerals in Venusian atmospheric conditions (e.g., Santos et al. 2023), in order to prepare for orbital infrared spectroscopic measurements (Morlok et al. 2019; Berger et al. 2019; Helbert et al. 2021; Treiman et al. 2021; see also the review in Widemann et al. 2023). Another kind of useful simulation would be laboratory analogs of the cloud droplets and surrounding atmosphere (facilitated by the fact that temperature and pressure conditions in the clouds are very close to Earth’s) to better assess poorly constrained heterogeneous reactions in particular.
